# Night duty and decreased brain activity of medical residents: a wearable optical topography study

**DOI:** 10.1080/10872981.2017.1379345

**Published:** 2017-09-27

**Authors:** Masaki Nishida, Senichiro Kikuchi, Fumikazu Miwakeichi, Shiro Suda

**Affiliations:** ^a^ Department of Psychiatry, Jichi Medical University, Shimotsuke, Japan; ^b^ Faculty of Sports Science, Waseda University, Tokorozawa, Japan; ^c^ Department of Rehabilitation Sciences, Gunma University Graduate School of Health Sciences, Maebashi, Japan; ^d^ Department of Statistical Modeling, The Institute of Statistical Mathematics, Tachikawa, Japan; ^e^ Department of Statistical Science, School of Multidisciplinary Sciences, Graduate University for Advanced Studies, Tachikawa, Japan

**Keywords:** Residents, self-perceived fatigue, night, shift, sleep, blood drawing, dorsolateral prefrontal cortex, actigraphy, wearable optical topography

## Abstract

**Background**: Overwork, fatigue, and sleep deprivation due to night duty are likely to be detrimental to the performance of medical residents and can consequently affect patient safety.

**Objective**: The aim of this study was to determine the possibility of deterioration of cerebral function of sleep-deprived, fatigued residents using neuroimaging techniques.

**Design**: Six medical residents were instructed to draw blood from artificial vessels installed on the arm of a normal cooperator. Blood was drawn at a similar time of the day, before and after night duty. To assess sleep conditions during night duty, the participants wore actigraphy units throughout the period of night duty. Changes in cerebral hemodynamics, during the course of drawing blood, were measured using a wearable optical topography system.

**Results**: The visual analogue scale scores after night duty correlated negatively with sleep efficiency during the night duty (ρ = −0.812, p = 0.050). The right prefrontal cortex activity was significantly decreased in the second trial after night duty compared with the first (p = 0.028). The extent of [oxy-Hb] decrease, indicating decreased activity, in the right dorsolateral prefrontal cortex correlated negatively with the Epworth sleepiness score after night duty (ρ = −0.841, p = 0.036).

**Conclusions**: Sleep deprivation and fatigue after night duty, caused a decrease in the activity of the right dorsolateral prefrontal cortex of the residents, even with a relatively easy routine. This result implies that the brain activity of medical residents exposed to stress on night duty, although not substantially sleep-deprived, was impaired after the night duty, even though they apparently performed a simple medical technique appropriately. Reconsideration of the shift assignments of medical residents is strongly advised.

**Abbreviations:** DLPFC: Dorsolateral prefrontal cortex; ESS: Epworth sleepiness scale; PSQI: Pittsburgh sleep quality index; ROI: Regions of interest; VAS: Visual analogue scale; WOT: Wearable optical topography

## Introduction

Residency training is an important opportunity for new physicians to gain knowledge and acquire clinical skills in their chosen specialty. However, most residents are subject to considerable psychological stress, fatigue, and sleep deprivation, which has a major impact on all aspects of their lives, including detrimental effects on learning and clinical performance [1]. Numerous studies have linked fatigue and sleep deprivation of resident physicians to poor performance in neurobehavioral testing, lack of motivation, and attentional failure [2,3]. Disturbed sleep not only reduces the quality of life, but also deteriorates the psychological well-being of resident physicians [4]. Alarmingly, these distressing conditions are associated with an increased risk of self-perceived major medical errors, including tacit incidents of critical medical accidents.

To prevent possible errors and reduce the risks to patient and physician safety, the Accreditation Council for Graduate Medical Education has imposed limitations on the working hours for U.S. medical residents restricting them to less than 320 hours in a four-week period [5]. In Japan, a large-scale survey indicated that the total working time of medical residents in a week was 68.0 ± 18.4 hours; however, 26.4% of the residents worked more than 80 hours [6]. Several studies have been conducted to determine the relationship between work and sleep hours and rates of medical errors among physicians. These studies have concluded that elimination of extended shifts decreased attentional failures during night duty [3,7].

Several hypotheses have been proposed to explain the reasons for decreased cognitive performance associated with sleep deprivation due to prolonged wakefulness. Cognitive impairments may be mediated predominantly through decreased alertness and attention that occurs with wake-state instability [8,9]. Of the large array of cognitive domains, the executive processes of working memory, which are of considerable importance for performing a medical technique, play a crucial role in certain attentional functions, such as sustained attention [10]. Thus, the deterioration in performance due to sleep deprivation would most likely be reflected in simple and monotonous tasks requiring reaction speed or vigilance.

Sleep deprivation affects the functioning of certain brain areas and thus impairs cognitive performance. Horne has suggested that sleep deprivation impaired particularly, the cognitive performances that are associated with the prefrontal cortex, including higher functions [11]. Wang et al. determined that the fronto-parietal connectivity has great importance in working memory with attentional control [12], which would be vulnerable to sleep deprivation. In addition, a number of neuroimaging studies that have focused on sleep deprivation have revealed inappropriate activation of predominantly the prefrontal regions, in the working memory system [13–16]. Nevertheless, the corpus of evidence has been limited to healthy normal volunteers; the brain alterations due to sleep deprivation in physicians working in hospitals have been poorly investigated.

The aim of the present study was to evaluate the alterations in the brain activity of sleep-deprived, exhausted physicians, after a night duty, by employing a wearable optical topography (WOT) system and a waist-worn actigraph, which are easily installed and prove less obstructive to the study participants as they perform their duties. To avoid individual differences due to the procedures of specific medical techniques, we decided to use the blood drawing procedure, a routine clinical technique, as the loaded task. We hypothesized that participants would experience fatigue and impaired sleep quality, despite maintaining the duration of sleep, and that their performance in the blood drawing task would not be affected, although cerebral activity after night duty would be impaired, even though performing a relatively simple routine procedure.

## Methods

### Participants

The study was conducted from April 2013 to March 2015. The study population included all postgraduate years 1 and 2 psychiatry residents from one university hospital. The total number of available residents was 40 residents. However, we eliminated N residents who did not meet the following inclusion criteria: (1) engaged in inpatient treatment for at least one month and (2) performed night duty at least twice. Exclusion criteria were (1) a history of sleep disorder or serious comorbid illness (i.e., diabetes, epilepsy); (2) current use of prescription sedative or stimulant; (3) not engaged in at least three night duty shifts during a 30-day period; and (4) right-handedness. The final sample of eligible volunteers was six (6/N residents who met inclusion criteria*100) psychiatry residents (four males, two females; mean age, 31.8 ± 4.2 years). After imparting comprehensive information pertaining to the goals of this study, written informed consent to the study protocol was obtained from all the study participants. The study protocol was carried out in accordance with the Helsinki Declaration, as revised in 1989, and was approved by the ethics committee of Jichi Medical University (protocol number 13–05).

Prior to each experiment, the participants were administered the Pittsburgh sleep quality index questionnaire to assess sleep quality and the Epworth sleepiness scale (ESS) to evaluate subjective sleepiness. The visual analogue scale (VAS) was used to measure fatigue in participants. In addition to the questionnaire and interview, blood pressure and heart rate were measured prior to the experiments.

### Experimental design

**Figure 1.**
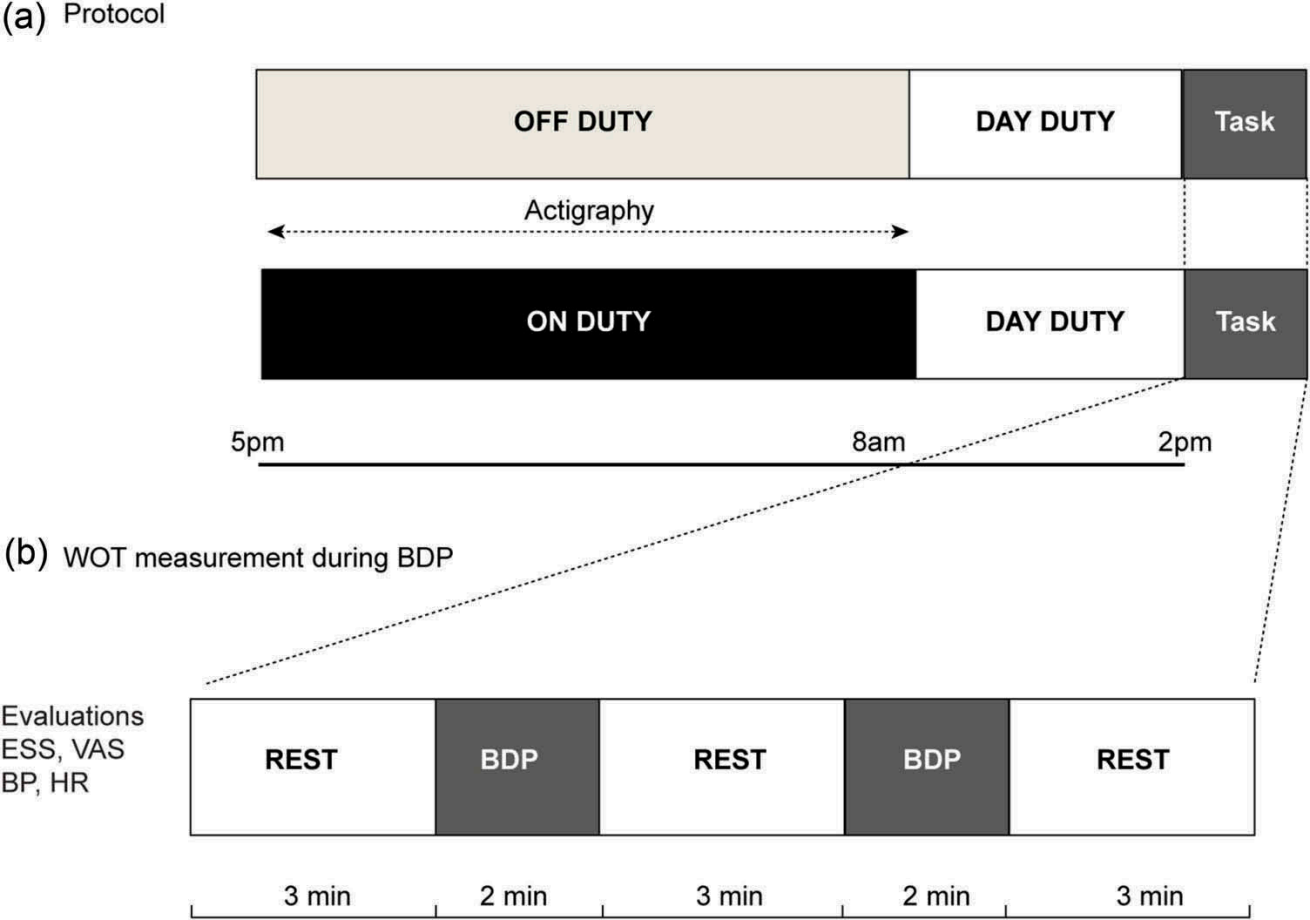
Experimental design. (A) Outline of the protocol for before and after night duty. (B) WOT measurement during blood drawing. Participants were instructed to limit movements three minutes before actual performance of blood drawing. Blood drawing procedure was performed twice, which was followed by a three-minute rest period. Participants were requested to perform the experiment both before and after night duty. BDP: Blood drawing procedure; ESS: Epworth sleepiness scale; VAS: Visual analogue scale for self-perceived fatigue; BP: Blood pressure; HR: Heart rate; WOT: Wearable optical topography.

A single center, randomized, crossover study design was used, as shown in the Figure 1 (A,B). Each participant was subject to two experimental conditions, one condition serving to assess the effects of night duty in the hospital, performed prior to the experiment, and the second without night duty, while at home, as a control apart. The two task conditions were administered in a counterbalanced order. The experiments started at 2 pm under both conditions.

Each experiment involved a one-condition block design, with a combination of control and stimulation blocks. After a preliminary three-minute control block, participants performed two trials of blood drawing, each over a two-minute period, followed by the control block, during which they were requested to limit their movements to avoid the inclusion of motion-derived artifacts.

### Night duty in the hospital

The participants were assigned night duty in the Department of Psychiatry of a university hospital. They were asked to start attending to calls (using the mobile phones) at 5 pm, from the psychiatric ward as well as the outpatient and emergency departments, until the night duty was terminated at 8:30 am on the next day. The participants usually stayed in the duty room, which was located in the ward. On completion of their night duty, they were instructed to report the number of phone calls received throughout the shift.

### Blood drawing procedure

**Figure 2.**
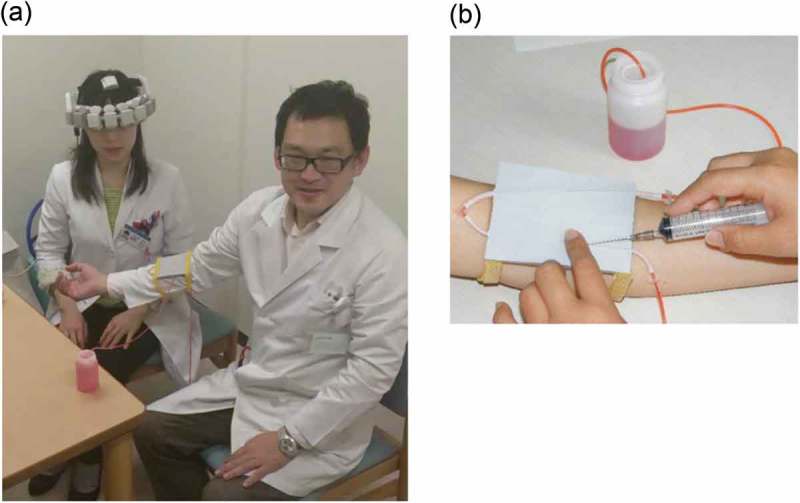
Experimental setup for wearable optical topography measurements during the blood drawing procedure. (A) A photograph of the measurement device. Participants wore the probe unit on their heads while drawing blood from the artificial vessels installed on the cooperator. (B) The imitation vessel apparatus (KAREN®, Fuso Runner Industry, Co., Ltd.).

Efficiency with the procedure of drawing blood, the most common technique performed by medical professionals, was tested using an experimental set-up designed to simulate a typical natural condition (Figure 2 (A,B)). Each session began after the WOT probes had been placed on the head (over the frontal regions) of the participants while they were seated in a comfortable chair, facing a cooperator. We installed a blood collection and intravenous injection training apparatus (KAREN; Fuso Rubber Industry Co., Ltd., Saitama, Japan) used for the training of medical professionals, on the arm of the cooperator. The apparatus comprising of artificial vessels embedded in a thin rubber pad, with red-inked water, representing blood, circulating in the vessels due to air pressure, representing the human arm with vessels, was constructed for testing the efficiency of drawing blood.

To measure baseline static cerebral activity, the participants were instructed to rest and limit their movements for three minutes, as required for a control block. On receiving the indication from the examiner, the participants started drawing blood from the vessels installed on the arm of the cooperator, repeating the procedure twice, each over two-minute periods, for the WOT measurements. After completing the blood drawing procedure twice, they were requested to rest and limit their movements for three minutes, as required for a control block. Subsequently, the participants were instructed to repeat the two-minutes blood drawing procedure.

The blood drawing procedure consisted of the following steps: (1) The participant wore gloves and placed a tourniquet around the arm of the cooperator; (2) The injection syringe was prepared and the skin of the cooperator’s arm was disinfected; (3) The syringe was then inserted into the artificial vessel and artificial blood was drawn; (4) The syringe was extracted and the artificial blood was transferred into a tube; and (5) The tourniquet was removed and the used items were discarded. During the experiment, the participants were instructed to limit their head movements as much as possible and maintain silence to avoid unnecessary noise.

The assessment of performance was based on whether the participants had successfully performed the blood drawing procedure, and the number of failures was counted. The speed of the blood drawing procedure was excluded from analysis due to individual differences of the participants with respect to the speed of performance.

### Actigraphy recording

The details of the actigraphy procedure were described in previous studies [17,18]. Briefly, a waist-worn actigraph (FS-750; Estera Corporation, Saitama, Japan) was used to record sleep parameters. The participants were instructed to wear the actigraph before going to bed, the night prior to the WOT evaluations, until they got out of bed. This small, light, rectangular device (external dimensions: 75  × 33.5 × 10.8 mm (width × height × depth)) is used to record the amount of activity with an internal three-axis accelerometer (electrostatic capacity sensor). The number of times the acceleration exceeds a reference value is summed, every 0.125 seconds, and the value is recorded as the activity value over two-minute bins. The activity intensity is calculated from the activity value on a scale from 0 to 31 (32 levels). An activity intensity of 0 indicates that the subject was stationery and the large values, high amount of activity.

An algorithm for the FS-750 actigraph that determines sleep and wakefulness was included in the SleepSignAct software (Kissei Comtec Co. Ltd., Matsumoto, Japan); the formula used and validation of the process for use in polysomnography (PSG) has been described in previous studies [17,18]. According to this algorithm, total sleep time was calculated as the amount of total time in bed after subtraction of the periods of wakefulness. Sleep efficiency was defined as the percentage of time the participant was asleep while in bed and was represented by the ratio of total sleep time to total time in bed.

### WOT measurements

**Figure 3.**
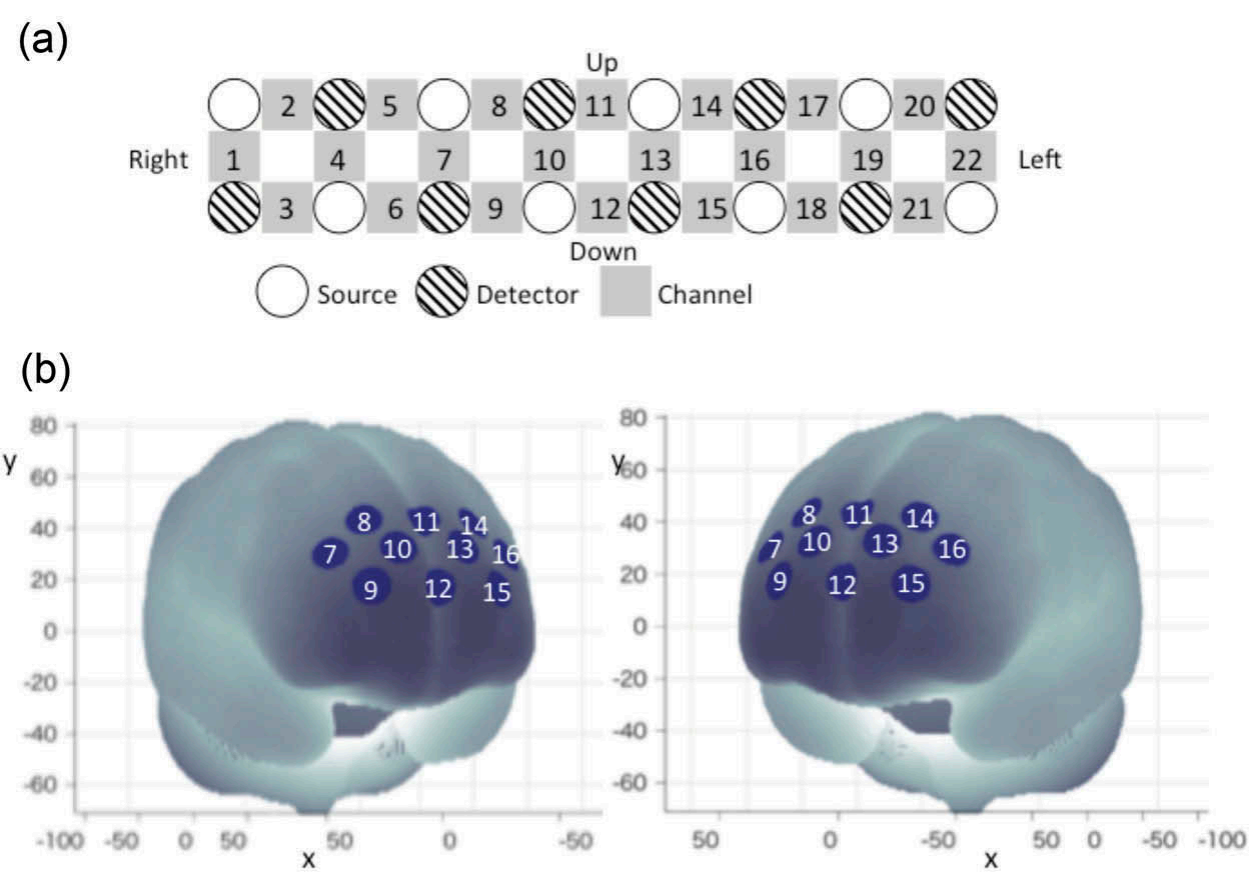
WOT setting and channel location. (A) Probe positions of lights sources and detectors and channel positions of WOT-100. Each channel position is defined as the center point between each light source and detector. (B) Measurement positions on a standard brain using the Montreal Neurological Institute coordinate system.

The details of the WOT-100 (Hitachi High-Technologies Corporation Co. Ltd., Tokyo, Japan) have been described in a previous study [19,20]. The probe unit has a 2 × 8 alternating arrangement of irradiation and detection positions covering the entire forehead, with 22 measurement points (Figure 3 (A)). Each pair of irradiation and detection units was separated by 30 mm, providing the probe unit with a coverage area of 30 × 210 mm^2^ on the participant’s forehead, including the bilateral temples, accumulating 5-Hz sampling data. This arrangement enables us to monitor the cortical activations mainly in the dorsolateral prefrontal cortex (DLPFC) and the rostral prefrontal area.

In this study, we used a modified WOT system that has laser diodes with two light sources of different wavelengths (754 and 830 nm) in the probe unit. Sato et al. have suggested that noise levels in hemoglobin (Hb) changes decreased when using wavelengths shorter than 782 nm paired with 830 nm, although the detected power appears to be weaker at the shorter wavelength [21].

To account for the variability of channel positions, probe positions were validated in previous studies by measurements of a three-dimensional digitizer, with a Montreal Neurological Institute coordinate system [22]. As shown in Figure 3 (B), data from 10 of the 22 originally used channels were selected for analysis whereas data from the remaining 12 channels (ch1–ch6 and ch17–ch22) were discarded due to contamination by motion artifacts during the blood drawing procedures. Ten channels (ch7–ch16) approximately corresponded to the inner bilateral regions of the DLPFC. Each channel detected oxy-hemoglobin [oxy-Hb] change as a time-series waveform at 0.2-s sampling intervals. A wireless connection was used to send the measured data to a computer in real time.

In addition, area of the prefrontal regions with the 10 channels was divided into two large regions of interest (ROIs): the left and right prefrontal regions, while discarding the temporal regions, with each ROI containing four channels (left frontal: ch13–ch16; right frontal: ch7–ch10).

### Data analysis

The acquired scores and actigraphic data of participants, obtained under the two conditions (before and after night duty), were compared using the Wilcoxon signed-rank test. To analyze the optical data, changes in the product of the concentration (*C*) and the effective optical path length (*L*) for oxy- and deoxy-Hb were calculated using the modified Beer- Lambert law [23].

We defined a three-minute resting period prior to blood drawing procedure as the pre-task resting period and a three-minute period after blood drawing procedure as the post-task resting period, respectively. The amplitude of the WOT signals were z-transformed using the averaged Hb signals (*ΔC*ʹ_oxy_, *ΔC*ʹ_deoxy_) and standard deviations during resting periods for each channel. After integrating [oxy-Hb] values obtained during the blood drawing procedure, differences in integrated [oxy-Hb] values during the blood drawing procedure for each channel were compared between the following three conditions: (1) before and after night duty; (2) two trials before night duty; and (3) two trials after night duty. Subsequently, differences in mean activated [oxy-Hb] values during the blood drawing procedure were compared between the two trials performed before night duty, as well as between the two trials performed before and after night duty. The mean [oxy-Hb] changes in ROIs were calculated under similar conditions as those of each of the channels.

Spearman rank-order correlation analysis was performed to examine the relationship between the psychometric questionnaire and measured physical parameters, actigraphic variables, and mean [oxy-Hb] changes for each channel. The false discovery rate-based procedure incorporated into the Benjamini–Hochberg method was adopted for multiple testing corrections of the analyses using data from 10 channels to avoid possible Type I error. Statistical analyses were performed using SPSS ver. 19.0J (IBM, Inc., Armonk, USA) and MATLAB R2015b (MathWorks, Inc., Natick, USA) software.

## Results

### Demographic characteristics

The demographic characteristics of the study participants are shown in Table 1. There were significant increases in the heart rate (p = 0.043) and VAS scores (p = 0.046) after night duty compared to those before night duty. The results indicated an increase in the ESS, which is related to daytime sleepiness, after night duty, although this increase was not statistically significant. The trials performed after night duty showed that the ESS significantly correlated with sleep efficiency during the night duty (ρ = −0.812, p = 0.050).

**Table 1.** Demographic characteristics of participants, behavioral data and sleep variables.

Demographics	
Age (years)	31.8 ± 4.2
Gender (female/male)	4/2
CES-D	12.0 ± 7.9
PSQI	4.8 ± 2.9

	Pre night duty	Post night duty	Z-Value	p-Value
Systolic blood pressure (mmHg)	118.5 ± 14.6	117.5 ± 11.1	0.524	0.600
Diastolic blood pressure (mmHg)	72.3 ± 10.1	76.3 ± 14.1	0.419	0.675
Heart rate (bpm)	76.2 ± 18.9	83.3 ± 15.3	2.022	0.043
ESS	6.5 ± 4.6	10.8 ± 3.4	1.677	0.046
VAS	3.2 ± 1.7	5.2 ± 1.8	1.992	0.093

	Pre night duty	Night duty	Z-Value	p-Value
Total sleep time (min)	327.7 ± 72.1	283.3 ± 57.5	0.944	0.345
Sleep efficiency (%)	77.3 ± 16.9	66.9 ± 13.7	0.944	0.345

CES-D: Center for epidemiologic studies depression scale; PSQI: Pittsburgh sleep quality index; bpm, beats per minute; ESS: Epworth sleepiness scale; VAS: Visual analogue scale. Data are presented as mean ± SD.

Of the sleep parameters evaluated by waist actigraphy, total sleep time and sleep efficiency during the night duty were worse as compared to before night duty; however, the differences between the two conditions and did not reach statistical significance (total sleep time: p = 0.345, sleep efficiency: p = 0.345).

In terms of performance measurement, one of the participants failed to perform the task properly after a night duty condition.

### Comparison of WOT signals before and after night duty

**Figure 4.**
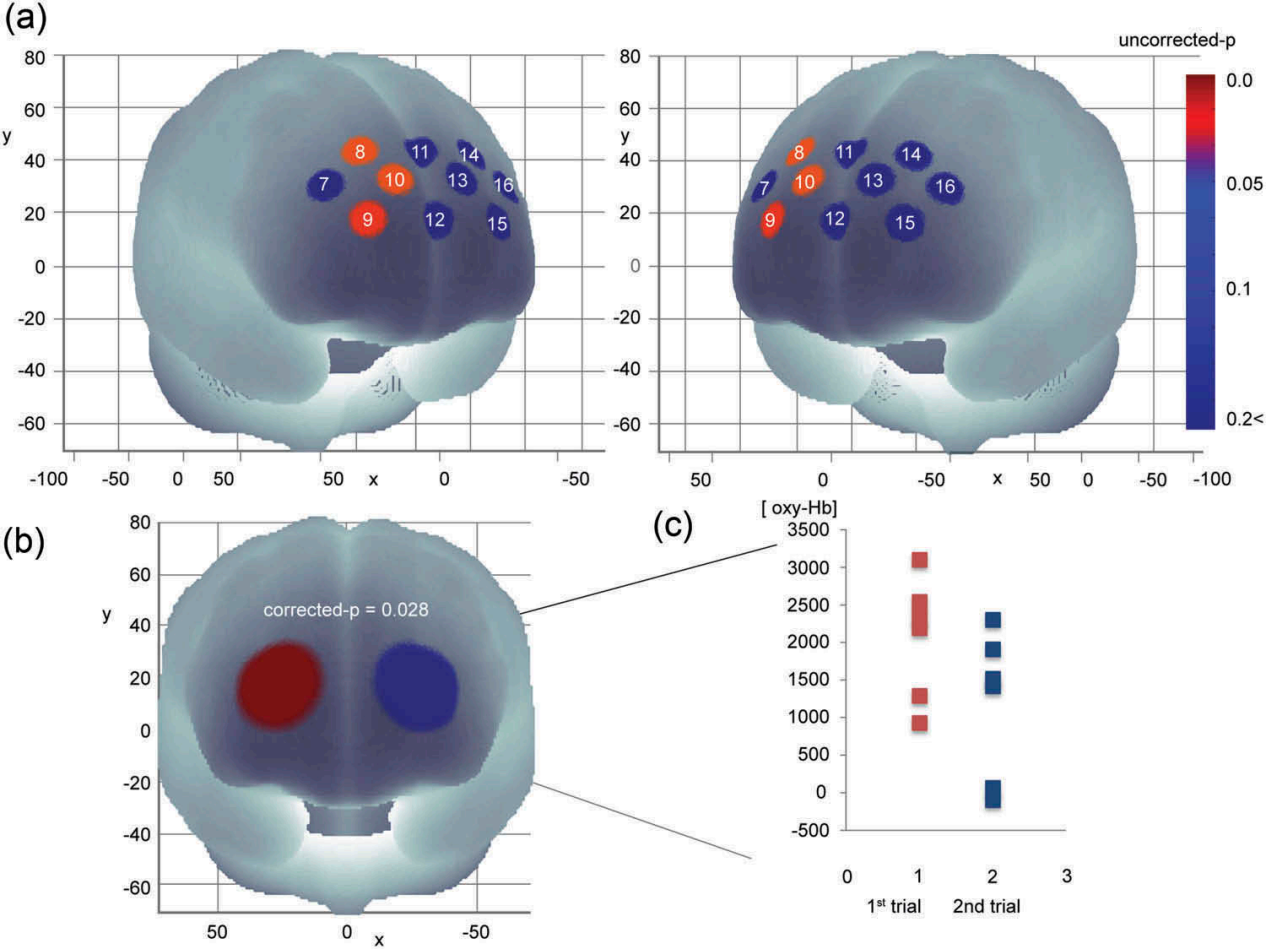
Results of comparison of mean [oxy-Hb] values during the blood drawing procedures between the first and second trials after night duty. (A) Channels in warm colors showing significantly decreased [oxy-Hb] changes during the blood drawing procedure in the second trial compared with those in the first (p < 0.05; uncorrected). (B) Regions of interest (ROI) of the left prefrontal regions showed significantly decreased [oxy-Hb] changes during the blood drawing procedure in the second trial compared with those in the first (p = 0.028; false discovery rate-corrected). (C) The raw mean [oxy-Hb] value of each ROI region in the first and second trials after night duty.

There was no significant difference in [oxy-Hb], which represents brain activity associated with the blood drawing procedure before and after night duty, as measured by WOT signals for the individual channels. The two trials before night duty showed no significant change in [oxy-Hb]. Although channels 8, 9, and 10 showed significantly smaller [oxy-Hb] values in the second trial after night duty compared to the first (uncorrected p = 0.046, 0.028, and 0.046, respectively) (Figure 4 (A)), the false discovery rate correction revealed that these differences were not significant. No other channel showed statistically significant differences in any of the comparisons related to the trials.

Despite the lack of significant differences in data among the various channels, analysis of ROIs showed a significant decrease in [oxy-Hb] in the right prefrontal region during the second trial after night duty (p = 0.028), whereas there was no significant change in the left prefrontal region (p = 0.173) (Figure 4 (B, C)).

### Relationship between WOT signals and variables

**Figure 5.**
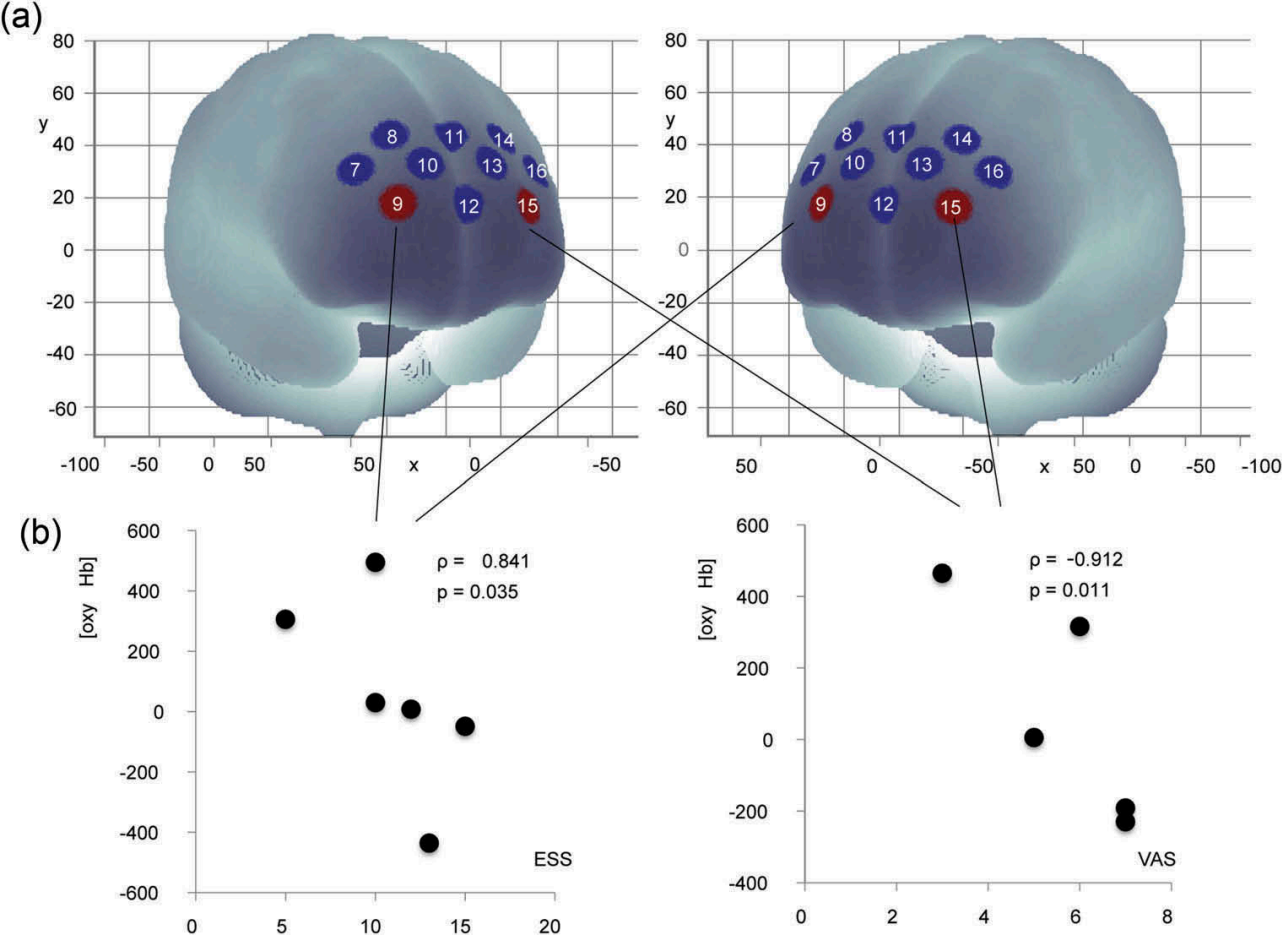
Correlations between mean [oxy-Hb] values and variables. (A) Correlation of mean [oxy-Hb] values during blood drawing procedure with Epworth sleepiness scale and visual analogue scale after night duty. Warm colored channels show significant correlation, as determined by Spearman rank-over analysis. (B) Scatterplots for correlations between channels 9 and 15. oxy-Hb: oxygenated hemoglobin signal; ESS: Epworth sleepiness scale; VAS: visual analogue scale.

The mean [oxy-Hb] for channel 9 for the second trial after night duty correlated negatively with ESS after night duty (ρ = −0.841, p = 0.036). In turn, the mean [oxy-Hb] for channel 15 for the second trial after night duty correlated negatively with the VAS scores after night duty (ρ = −0.912, p = 0.011) (Figure 5 (A,B)).

## Discussion

Through analysis of the WOT data, we found that night duty affected brain activity even during a relatively simple, routine procedure, such as blood drawing. This was confirmed through our observation of the decrease in [oxy-Hb] of the right prefrontal region when drawing blood before and after night duty. Although an increasing number of studies have investigated the effects of sleep deprivation on cognitive and executive function performance associated with brain alterations, analyses have been limited to normal healthy participants [13,24,25]. Furthermore, these studies were less likely to involve psychological stress, such as that related to real-time night duty experience, other than that associated with laboratory conditions of sleep deprivation. To the best of our knowledge, the present study is the first attempt of an evaluation of alterations in the brain functions of on-duty medical residents by direct assessments of cerebral hemodynamic reactivity using a noninvasive WOT method.

The actigraphic recordings indicated that the participants were able to maintain sleep quality while on-call, comparable to that achieved at home. This may be attributable to the fact that night duty in a psychiatric ward mainly consists of attending to phone calls in the duty room, which is substantially different from the demands of departments involving increased and rapid activity, such as the emergency room or intensive care unit. However, the inverse correlation between the VAS scores and sleep efficiency during night duty implied that a reduction in sleep quality, although small, was likely to subsequently increase the extent of perceived daytime fatigue. An activated autonomic response, represented by an increase in the heart rate, due to night duty is observed [26,27]. Thus, we speculate that the residents endured considerable psychological stress when receiving phone calls during night duty.

Previous studies, that focused on various surgical procedures, found no association between sleep deprivation and surgical outcomes or complications [28–31]. In contrast to a routine clinical procedure, such as blood drawing, surgical procedures require considerable attention and concentration, which help to overcome the feeling of fatigue and sleepiness due to night duty. Unlike surgical procedures, blood drawing is a routine clinical procedure; however, it proved to be difficult for our participants to maintain their attention and concentration.

The DLPFC area is involved in attention regulation that is mediated by neural activity in the frontal regions [32]. Previous studies have indicated that the right frontal lobe plays an important role in sustaining attention [33]. Sleepiness during the blood drawing procedure after night duty correlated negatively with the right prefrontal activity, suggesting right prefrontal involvement in attention and in working memory, which is defined as the temporary maintenance and manipulation of information necessary for performance [34]. Homma et al. demonstrated that the right prefrontal activity reflects the ability to overcome sleepiness during working memory tasks using an n-back task [35]. Despite unproven causality, a reduction in right prefrontal activity is closely associated with sleepiness and fatigue, perceived after night duty, and additionally modulated by emotional dysregulation [36].

With respect to the relationship between subjective fatigue and sleepiness, Suda et al. showed that decreased activities in the lateral frontal and superior temporal cortices were related to fatigue, although there was no association with sleep duration [37]. Ishii et al. reported that the left frontal cortex is involved in the evaluation of mental fatigue levels [38]. We assume that the fatigue perceived by the participants of our study was related to sleepiness due to poor sleep efficiency, thereby altering the complex network of the bilateral prefrontal cortices. Considering that the WOT system is unable to detect brain activity within the limbic and paralimbic systems, alternative neuroimaging studies are warranted to elucidate the effects of fatigue and sleepiness on the neural networks.

The primary limitations of this study involve the small sample size and low degree of control on the behavior of participants during the night duty, as well as the variability in the number of the calls received during night duty. The study involved the measurement of numerous parameters in a small sample, which resulted in substantial compromises in data analysis, although non-parametric statistics was applied. The small sample was partially due to the difficulty we faced in enrolling resident physicians for this study; a larger study cohort is necessary to confirm our findings that night duty is detrimental to brain function. Although the regulation of the behavior of the participants was inadequate when compared to a controlled sleep deprivation study, we believe that we replicated true psychological stress through real-time experience of night duty. Further studies focusing on endocrinological factors are required to examine the relationships between stress, fatigue, and sleepiness.

In conclusion, the cerebral activity in the right prefrontal region was decreased significantly during the blood drawing procedure performed after night duty. Although there was no deterioration in performance or obvious complications with a routine technique such as blood drawing, measures of fatigue and daytime sleepiness were significantly correlated with the decreased cerebral activity. This suggests that attention deficits due to the combined effects of fatigue and sleepiness could have a deleterious impact on resident physician performance after night duty. The shift assignments of medical residents should be re-evaluated to prevent medical complications and maintain the well-being of physicians.
